# Supplementary figures and images for: Developing and Testing a Bayesian Analysis of Fluorescence Lifetime Measurements
Source: PLoS One. 2017 Jan 6;12(1):e0169337. doi: 10.1371/journal.pone.0169337 (PMC5217968; doi:10.1371/journal.pone.0169337)

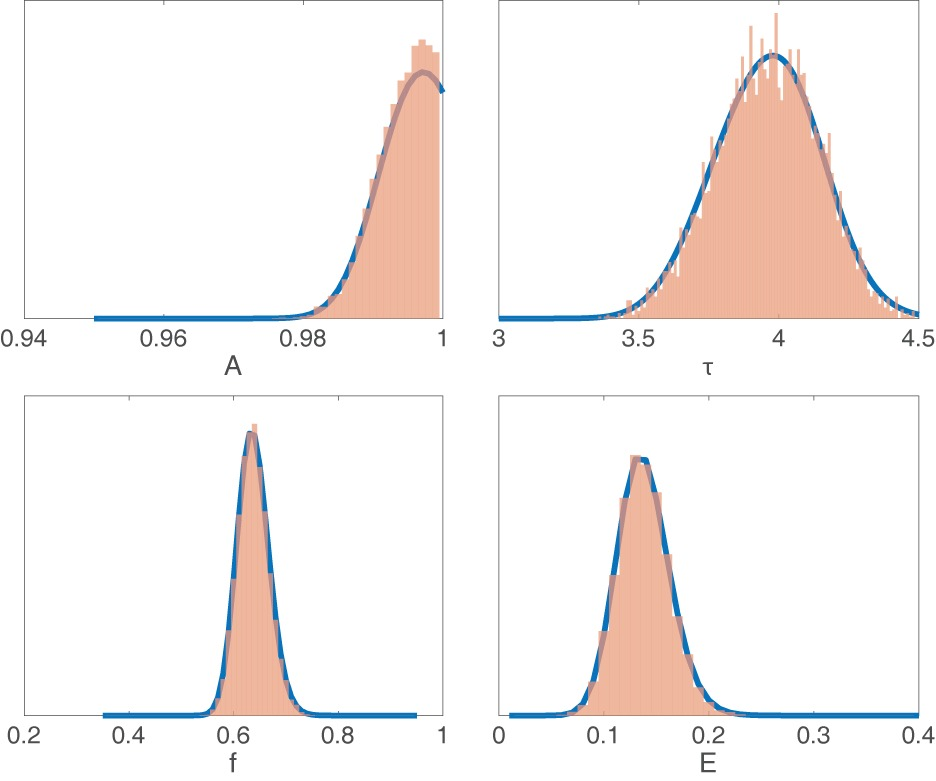

Supplement: S1 Fig — Results from Markov chain Monte Carlo (red) and grid points (blue) were generated from the same data set. (TIF) [file pone.0169337.s001.tif]
