# Supplementary material for: Developing and Testing a Bayesian Analysis of Fluorescence Lifetime Measurements
Source: PLoS One. 2017 Jan 6;12(1):e0169337. doi: 10.1371/journal.pone.0169337 (PMC5217968; doi:10.1371/journal.pone.0169337)
Supplement: S1 Appendix — (PDF) [file pone.0169337.s002.pdf]

S1 Appendix: Removal of Time Bins

A limitation of the Becker & Hickl system used here is that it cannot record photon arrival times for the entire collection period. In addition, time bins can be artificially removed from analysis to preferentially remove contaminating factors neglected in the analysis. For example, biological samples such as *Xenopus laevis* oocyte extracts can contain endogenous fluorophores with a lifetime significantly shorter than the donor lifetime. Photons collected from these endogenous fluorophores have a large contribution to early time bins and thus removal of these bins preferentially removes their effects.

While not utilized in the data presented here, removal of time bins can be taken into account in the likelihood function constructed above, Eqn 5, by weighting the population fractions, f_i by,

$$a = \frac{\sum^{kept\ time\ bins} p_{em,S}(t \in b_i | f_S, \tau_S)}{\sum^{all\ time\ bins} p_{em,S}(t \in b_i | f_S, \tau_S)}$$

$$b = \frac{\sum^{kept\ time\ bins} p_{em,L}(t \in b_i | f_L, \tau_L)}{\sum^{all\ time\ bins} p_{em,L}(t \in b_i | f_L, \tau_L)}$$

$$c = \frac{\sum^{kept\ time\ bins} p_B(t \in b_i | f_B)}{\sum^{all\ time\ bins} p_B(t \in b_i | f_B)}$$

$$D = f_S a + f_L b + f_B c$$

Such that,

$$p(t|\theta) = \prod_{i=1}^{N} \Big[\frac{a}{D} f_S \times p_{em,S}(t \in b_i | f_S, \tau_S) + \frac{b}{D} f_L \times p_{em,L}(t \in b_i | f_L, \tau_L) + \frac{c}{D} f_B \times p_B(t \in b_i | f_B) \Big]^{P_i} \quad (1)$$
